# The Recognition of the Micro-Events in Cement Composites and the Identification of the Destruction Process Using Acoustic Emission and Sound Spectrum

**DOI:** 10.3390/ma13132988

**Published:** 2020-07-04

**Authors:** Dominik Logoń, Krzysztof Schabowicz

**Affiliations:** Faculty of Civil Engineering, Wrocław University of Science and Technology, Wybrzeże Wyspiańskiego 27, 50-370 Wrocław, Poland; krzysztof.schabowicz@pwr.edu.pl

**Keywords:** quasi-brittle cement composites, acoustic emission, acoustic spectrum, micro events

## Abstract

This paper presents the recognition of micro-events and their concentration in quasi-brittle cement composites and the identification of the destruction process based on acoustic emission and sound spectrum. The tests were conducted on a quasi-brittle composite of a cement paste reinforced with a high volume of dispersed polypropylene fibers. The possibility of identifying the destruction process based on acoustic emission and sound spectrum was confirmed. This paper focused on the identification of micro-events using the 3D spectrum. It was shown that the identification of the concentration of micro-events precedes the occurrence of critical crack f_cr_, ending the Hooke’s law range. The ability to recognize this phenomenon with the use of the 3D spectrum makes it possible to predict the structure destruction process and subsequently to assess the structure destruction (micro and macro-cracks) and the reinforcement destruction (pull-off, breaking). It was confirmed that the three-dimensional spectrum provided additional information, enabling a better recognition of micro and macro-changes in the structure of the samples based on the analysis of sound intensity, amplitudes, and frequencies.

## 1. Introduction

Acoustic emission (AE) is a method that has been used for a very long time in concrete engineering [1]. Acoustic emission measurements can be applied to recognize the early hydration of cement [2,3]. Most papers have focused on determining the destruction process (cracks, maximum load, and failure of reinforcement in cement composites) [4,5,6,7,8,9,10,11,12,13,14,15,16,17,18,19,20,21,22,23,24,25,26,27,28,29,30,31,32,33,34,35,36,37,38,39,40]. It was noticed that AE is an effective method in determining the critical stress f_cr_ of cement composites corresponding to the first crack [4,5,6]. It is used for the accurate definition of the elastic range corresponding to Hooke’s law [7,8]. The continuous AE evaluation in composites and acoustic emission AE events sum (sum AE) has been applied to determine the first crack [4,9,10], micro and macro-cracks, and their propagation in the fracture process in cement composites with [11,12] and without reinforcement [13,14].

The test results indicate that AE is a good method for crack formation monitoring in mechanically loaded traditional [15,16] and high strength [17,18] cement composites. It was demonstrated that this method is effective during compression [19,20] and bending tests [21,22].

Acoustic emission measurements also focus on the possibility of identifying crack orientation [23,24,25] q, thus enabling the recognition of cracks occurring as a result of compression, tensile, and shear stress.

AE is used to identify the destruction process of different materials (e.g., reinforced geopolymer mortars) [26]. The effectiveness of acoustic emission measurements in structures control was confirmed [27,28,29]. This method is used for example to monitor and control bridges condition [30,31]. The AE is still improved for the purpose of the identification of failure processes in different materials and structures [32,33,34].

Our previous own works have focused on the correlation between AE and the individual failure processes of each of the different composite components based on the sound spectrum [7,35,36]. The conclusions indicate that for the accurate recognition of composite failure processes, the AE recording should be expanded to include the analysis of each sound separately (also a single signal in a very small range of frequencies) and the analysis of the range of sounds corresponding to a given mechanical effect with the use of acoustic spectrum. It was noticed that the acoustic spectrum 2D and 3D should be correlated with the load-deflection curve and with other acoustic effects, which enables the identification of the failure process.

The presented paper confirms that there is a possibility of correlation between AE and the failure process in quasi-brittle cement composites. This correlation enables a determination of the stage of damage in cement composites, increasing the safety of using the composite and a decision whether or not the damaged composite can be repaired.

The main innovation of this research is the possibility of identifying AE micro events in the area preceding the occurrence of critical crack initiating the destruction process in cement composites. The main idea in this paper is the possibility of micro event recognition in the Hooke’s law range, which enables the prediction of the destruction process.

The paper presents the results of our own studies on a selected paste sample in a four-point bending tensile test conducted at IPPT PAN in Warsaw, taking into consideration the current recommendations [37,38,39,40,41].

The results of tests carried out on a number of cement composites (cement paste, mortar, concrete, with and without dispersed reinforcement [40]) confirmed the general conclusions presented in the paper, indicating the possibility of their generalization.

## 2. Testing

### 2.1. Materials Used for Tests

High-strength cement composites w/(c + Sf) = 0.31, Portland Cement CEM I 42.5R (c), silica fume S_f_ = 10%c, siliceous fly ash 20%c, superplasticizer Sp, tap water (w).

Synthetic-structural polypropylene fibers (ASTM C-1116): density 0.91 kg/dm^3^, f_t_ = 620–758 MPa, E = 4.9 GPa, l = 54 mm, d = 0.48 mm, l/d = 113.

The specimens were reinforced with a polypropylene fiber volume of V_f_ = 5%. The samples with dimensions 40 × 40 × 160 mm were cut out from pre-formed slabs. Each beam was turned by 90° and cut to the depth of 7 mm.

### 2.2. Preparation of Specimens for Tests

**Figure 1:**
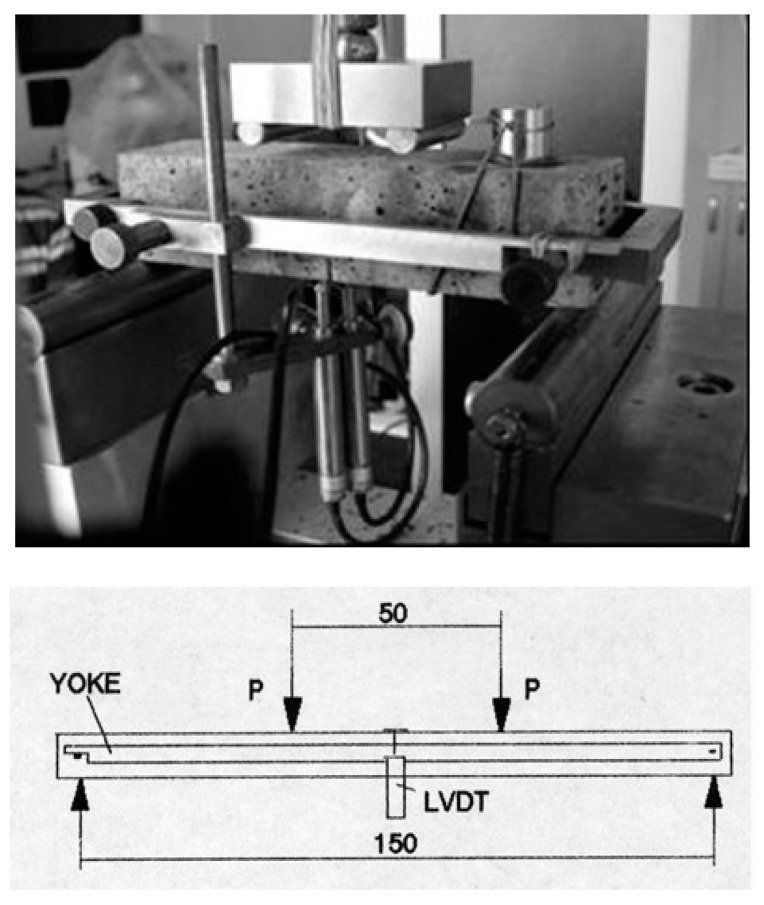
Four-point bending test [40].

Four-point bending tensile tests were carried out at IPPT PAN in Warsaw [40]. The measurements were conducted on sample beams with the dimensions of 40 × 40 × 160 mm (Figure 1). In the middle of the span, in the lower part, a cut (7 mm deep) was made in the specimens, in accordance with American Society for Testing and Materials ASTM 1018 [37]. This paper presents the results for quasi-brittle cement composite of paste with high volume of polypropylene fiber reinforcement.

### 2.3. Description of the Test Stand

The tests were conducted on three specimens. The results obtained for each of the three specimens separately confirm the conclusions contained in the paper.

**Figure 2:**
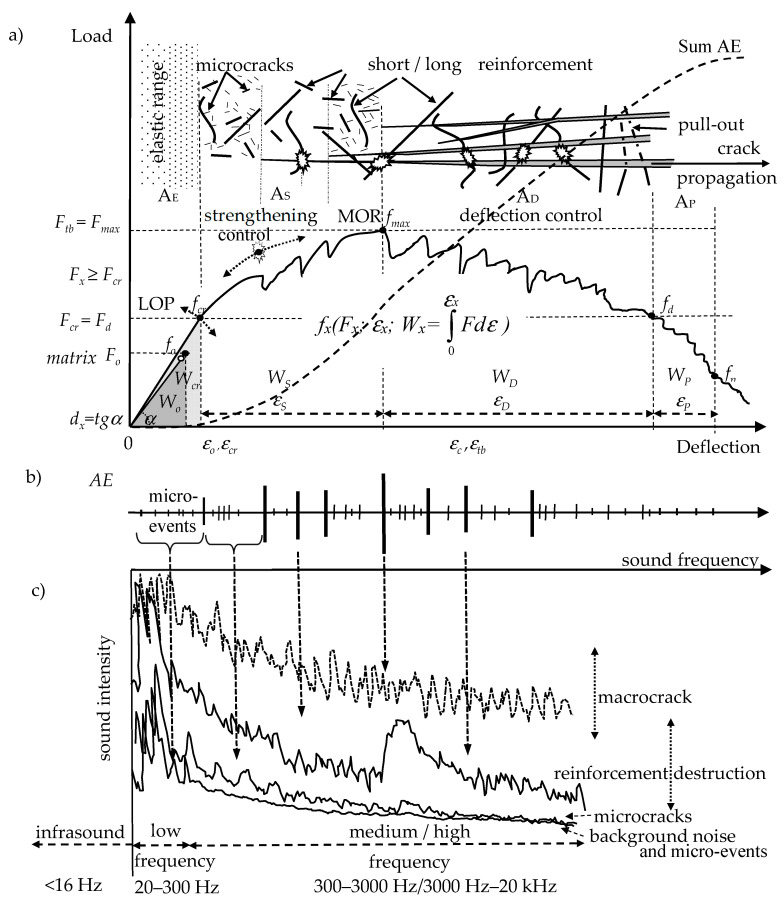
The quasi-brittle composite: (**a**) load-deflection curve, (**b**) AE, acoustic emission effects, (**c**) 2D acoustic spectrum (frequency amplitude depending on sound intensity) based on [36].

The loading process was carried out with a controlled, constant displacement speed equaling 0.05 mm/min. Deflection was recorded by means of two LVDT sensors located under the beam, using a “Yoke” clamp (Figure 2), [40]. During the test, the bending load and deflection of the specimen were measured. The testing procedure corresponded to the requirements of the ASTM C 1018 standard.

At the end of the test, a 5 mm deflection of the specimens was recognized, which was determined in relation to the neutral axis. The acoustic emission sensor was fixed to the top surface of the beams with the use of an elastic band and the surface was coated with coupling graphite grease (Figure 1).

The broadband AE sensor manufactured by Physical Acoustic Corp. enabled the recording of the AE signal within the frequency range of 10–1000 kHz.

The AE signal was recorded with the use of the ADLINK 9112 card with the sampling rate of 88.2 kHz, the 12-bit resolution, and the function of a continuous recording on a computer disk. Source files were saved in the format (.wav), which makes it possible to listen to the recorded signal with the use of the computer sound card speakers.

Thanks to the recorded data, it is possible to plot a load-deflection curve, record AE events, and aggregate them (total AE).

The acoustic emission effects were presented as a 2D and 3D acoustic spectrum (amplitude of the frequency depending on sound intensity). The 2D sound spectrum was achieved with the use of the Audacity program and the 3D spectrum using SpectraPLUS-SC (Pioneer Hill Software LLC, Poulsbo, WA, USA).

The quasi-brittle cement composites (ESD—Eng. elastic range, strengthening control, deflection control) were characterized by higher load and absorbed energy in the elastic range compared to the sample without reinforcement (Figure 2) [35]. The reinforcement effects may be presented by characteristic points *f_x_* (*F_x_*-load, *ε_x_*-deflection, *W_x_*-work) and areas A_X_ under the load-deflection curve.

Figure 2 presents the mechanical effects of the quasi-brittle cement composites with the corresponding acoustic effects and compiled acoustic spectra with various amplitudes corresponding to different mechanical effects (reinforcement breaking, pull-out, macrocracks, microcracking, and micro-events).

## 3. Test Results

**Figure 3:**
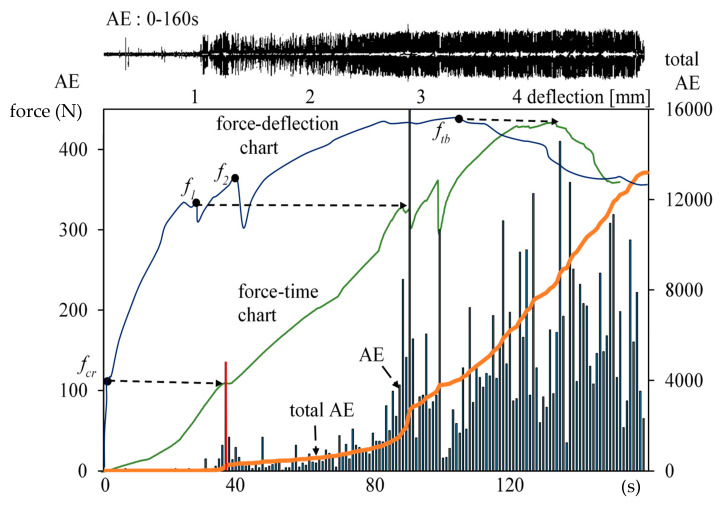
Results of tests on the composite in the range of 0–160 s: force-deflection, force-time curve, total acoustic emission AE, events, and AE recording.

**Figure 4:**
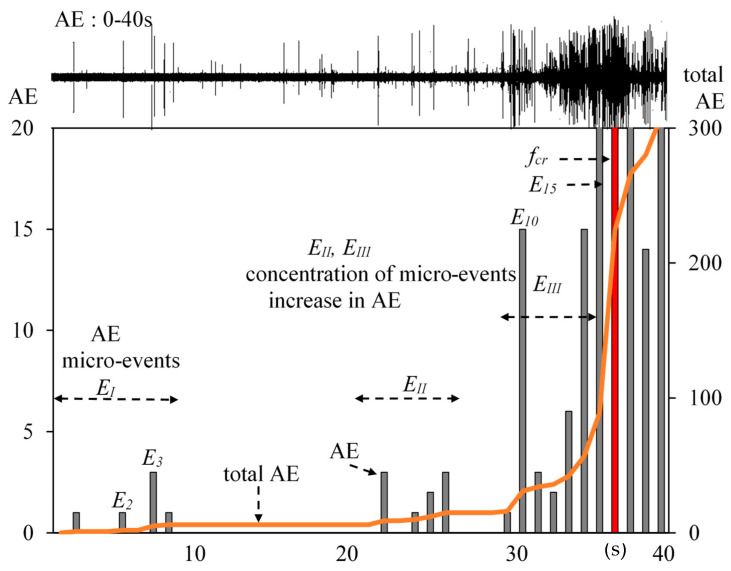
Results of tests on the composite Figure 1 in the range of 0–40 s: total AE, events, and AE recording.

The results are presented using the example of a quasi-brittle cement composite of slurry type with dispersed reinforcement in the form of structural polypropylene fibers (Figure 3 and Figure 4).

In the top part of Figure 3, the recording of AE effects is presented. The figure shows charts from four-point bending tensile tests, force-deflection, and force–time; in addition, it shows the recorded events as a function of time (0–160 s) and the AE events total.

The characteristic points and ranges of AE events are highlighted in the presented figure. In addition, the chart of total AE is shown. The red color marks the event f_cr_ (the end of Hooke’s law), corresponding to the critical point f_cr_ on the force-deflection curve. Point f_1_ corresponds to the sudden drop in stress in the strengthening area, and point f_tb_ corresponds to maximum stress.

In order to analyze the destruction process in the proportionality area shown in Figure 3, the effects obtained in that range of deflections (in the range of 0–40 s) were enlarged and presented in Figure 4.

**Figure 5:**
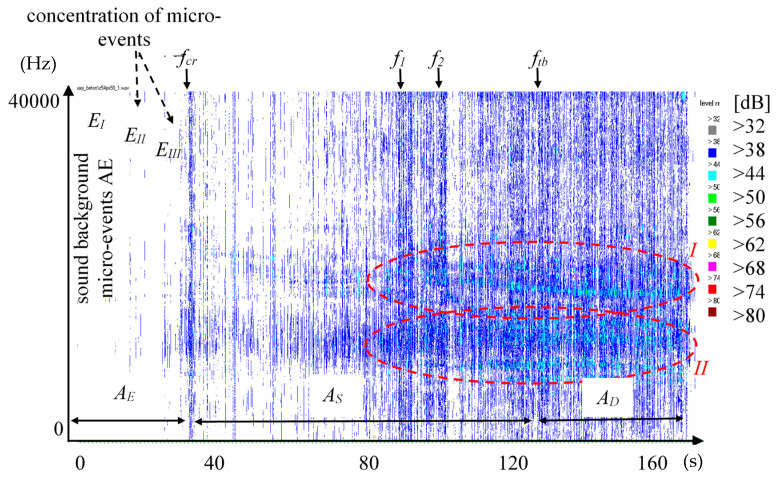
Spectogram of the AE signal.

The spectrogram of the AE signal of the tested composite is presented in Figure 5. Events are visible as vertical lines with marked characteristic points f_cr_, f_1_, f_tb_, and image of events occurring in quasi-brittle composites, with highlighted areas of proportionality A_E_, strengthening control A_S_ and deflection control A_D_. The area with a dominating impact of event noise as well as the areas with the concentration of events E_II_ and E_III_ are marked. The spectrum is presented in the range of low, medium, and high frequency from 0–4000 Hz. The red intermittent line in the spectrogram marks an area that is linked to the possible occurrence of dispersed reinforcement breaking. Figure 5 enables the observation of the concentration of micro-events preceding f_cr_ (E_II_ and E_III_).

**Figure 6:**
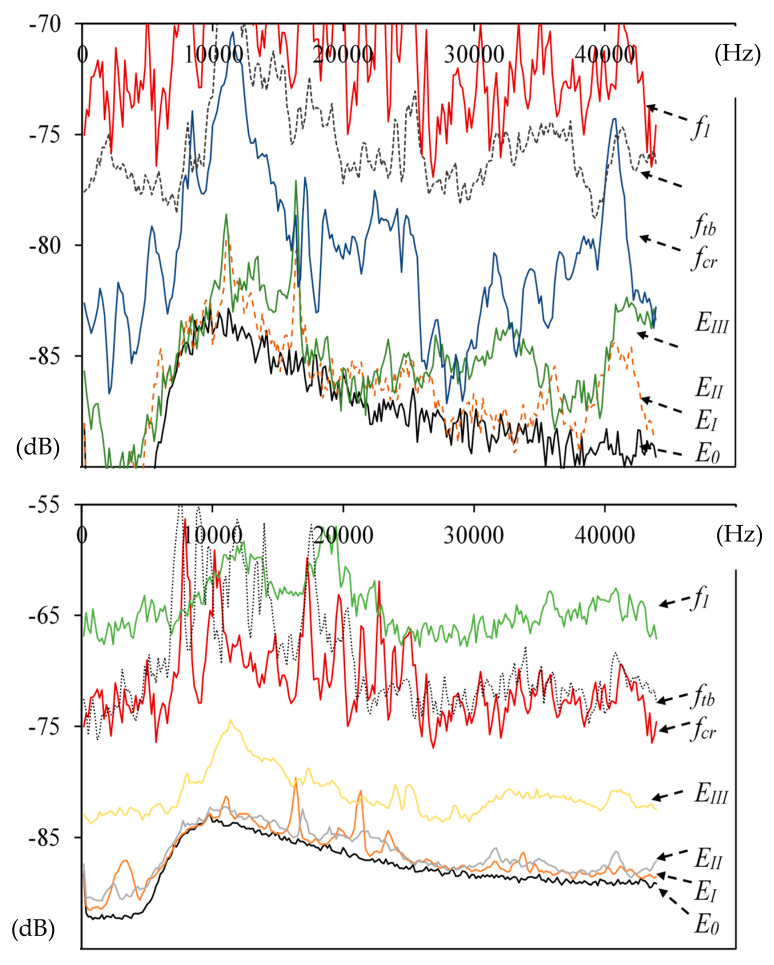
Sound spectra for characteristic events: sound background E_0_ and ranges of events: E_I_, E_II_, E_III_.

Figure 6 presents the 2D sound spectra for different AE effects obtained at characteristic points or ranges of events by means of the Audacity program. Event E_0_ corresponds to the background spectrum. Three characteristic areas were identified: E_I_, E_II_, and E_III_. Area E_I_ groups single events from E_1_ to E_4_, area E_II_ includes events from E_5_ to E_8_, and E_III_ refers to events from E_9_ to E_15_. The spectra are presented in the wide range of low, medium, and high frequency from 0–45,000 Hz.

**Figure 7:**
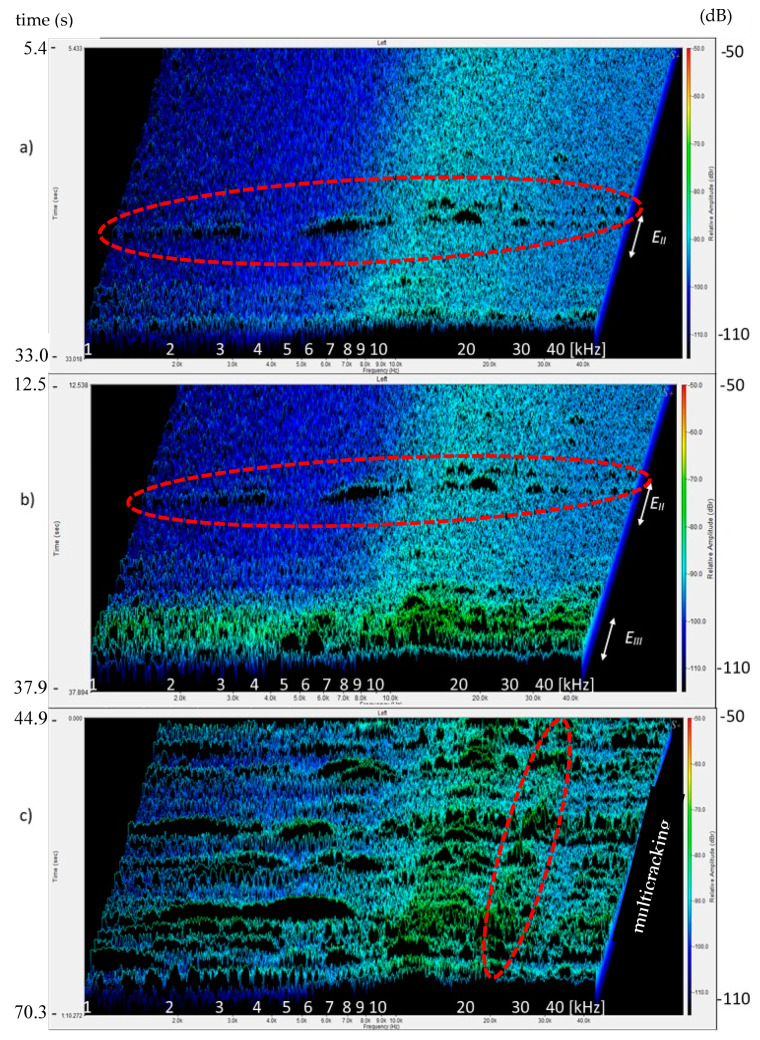
Composite sound spectra: (**a**) background, micro-events E_II_, (**b**) micro-events E_II_ and E_III_, (**c**) multicracking between points f_1_ and f_2_.

Figure 7a,b presents the 3D sound spectra from the Hooke’s law range. Figure 7a shows the sound background spectrum and spectra of single AE events (groups of sounds E_II_), with spectra in the time range of 5.4–33 s. Figure 7b presents the background spectra, and events spectra E_II_ and E_III_ in the time range 12.5–39.7 s. Figure 7c displays the background spectra and spectra of multicracking between points f_cr_ and f_1_ in the time range 44.9–70.3 s. Relative amplitudes (of sound intensity components) were analyzed between 50–110 dB. The range of sound intensity components for background spectra and spectra of events E_II_ was from −110 to −85 dB, while the group of events E_III_ and multicracking corresponded to the level of −80 dB.

**Figure 8:**
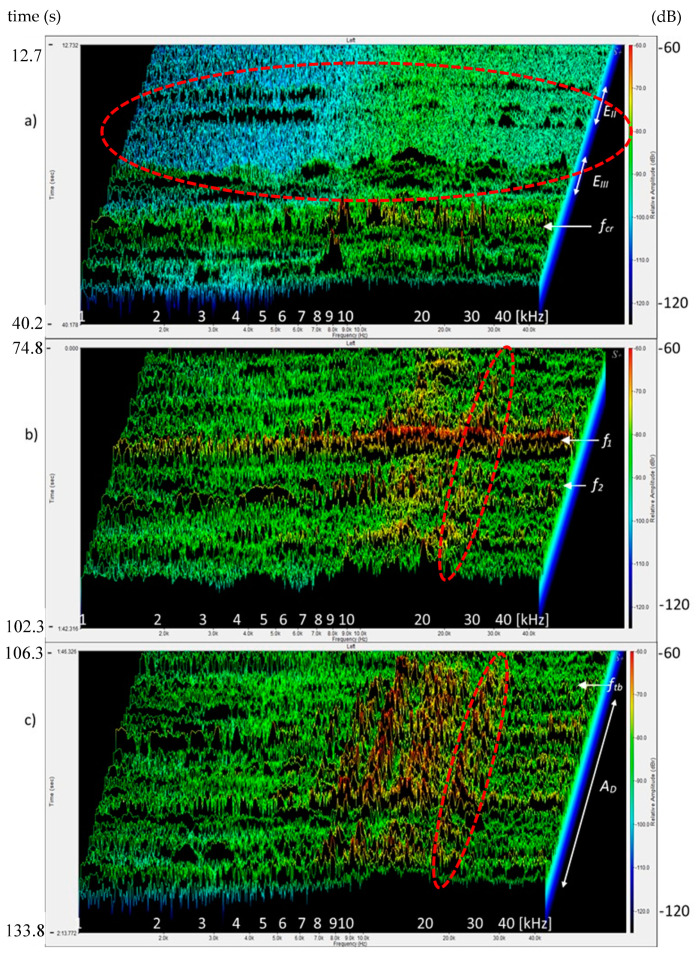
Composite sound spectra: (**a**) background, micro-events E_II_, E_III_, and f_c_, (**b**) event f_1_, (**c**) event f_tb_ and deflection control area A_D_.

Figure 8a is an image of the sound spectra of the composite: its background, events E_II_, E_III_, and the critical point f_cr_. Figure 8b presents the spectra of the area of sudden drops in stress in points f_1_ and f_2_ in the time range 74.8–102.3 s. Figure 8c shows the spectra of event in point f_tb_ and spectra of the deflection control area. The image is presented in the range 106.3–133.8 s. Relative amplitudes (sound intensity components) were analyzed in the range between 60–120 dB. The range of sound intensity components for background spectra and spectra of events E_II_ was from −110 to −85 dB, while the group of events E_III_ and multicracking corresponded to the level of −80 dB.

## 4. Discussion of the Results

Figure 3 presents force-deflection and force-time correlation obtained during bending–tension tests of a quasi-brittle cement composite with dispersed reinforcement. Presentation of these two charts in one figure enables a better recognition of the destruction processes. The force-deflection curve enables the identification of the proportionality, strengthening, deflection control, and crack propagation areas. The sudden decreases in the ability to carry stress that are recorded on the force-deflection curve indicate the appearance of macro-cracks/fractures including fiber breaking.

The force–time curve allows one to indicate the effects occurring at the same time as the recorded AE effects and the AE totals, which should be referred to the force-deflection curve. In order to precisely assess the composite destruction process, it is necessary to correlate both force-deflection and force–time curves with the measured AE effects. The recorded acoustic effects in the proportionality area is presented in 4, Figure 7b and Figure 8a. The obtained data indicate the recording of signals and AE micro events in the proportionality area AE. The measured AE effects have been linked mainly to the sound background signal occurring during the tests and to the occurring events. In the figures, AE events can be observed that have been linked to the individual microcracks occurring within the area of the Hooke’s law and micro-regrouping in the structure (micro-relaxation), which do not affect the stress-deflection linear correlation. The occurrence of microcracks before the occurrence of f_cr_, has been confirmed in other publications [20,26,36].

A micro-event precedes the occurrence of critical point f_cr_. As shown by Figure 4, Figure 5, Figure 6, Figure 7 and Figure 8, in the initial period of the operation of Hooke’s law, in addition to the background noise, single micro-events/signals appear. The spectra that correspond to sound backgrounds are characterized by the smallest relative amplitude (Figure 6 and Figure 7). Slightly larger intensities of the sound component correspond to micro-events, which do not affect the stress-deflection linear relationship. If the correlating spectra do not occur in groups and there is no increase in sound intensity components, then they may be considered as insignificant (Figure 7a).

The ability to identify them makes it possible to avoid the catastrophic destruction process in traditional cement composites and in quasi-brittle composites to avoid exceeding f_cr_. As is shown by the presented data, the sound spectrum corresponding to f_cr_ is characterized by the largest intensity of the sound spectrum component, which ends the concentration of events (Figure 6b and Figure 8a).

Recognition of the destruction process by means of the 3D spectrum has already been presented earlier [36] with respect to various cement composites and has also been confirmed in this paper.

What has been observed in this study is a clear division of relative sound amplitudes at the level of circa 7 kHz. Medium- and low-frequency sounds are characterized by a lower intensity of sound spectrum component compared to high-frequency sounds.

The analysis of the low-frequency spectra was not clear (it results from the measurement range of the head recording the sound), which is why it was disregarded in the presentation of the 3D image (Figure 7 and Figure 8). The analysis of sound spectra was conducted in the range of 1–40 kHz.

After exceeding f_cr_, the multicracking effect was observed in the tested composite, resulting in the appearance of the intensities of sound components stronger than the background spectra and single events in the proportionality area, but significantly smaller than the spectrum of f_cr_ (Figure 7c). The appearance of a macrocrack at point f_tb_ and the deflection control process A_D_ resulted in spectra with the sound intensity similar to f_cr_ with larger amplitudes in the range of 8–20 kHz (Figure 6, Figure 7 and Figure 8).

Figure 5 shows a 2D spectrogram that presents the possibility of the identification of destruction processes. It is a method of data imaging used previously by researchers. The obtained image does not show the velocity of increase in the relative amplitudes and contains too large ranges of amplitudes, which may contain various signals, especially those with small amplitudes, which makes it difficult to recognize them (especially with respect to single events and the phenomenon of their concentration). The spectrogram enables the recognition of AE effects with large differences in amplitudes. The use of frequency ranges makes it difficult to identify which area is responsible for the reinforcement breaking, and which is responsible for the pull-out of the reinforcement from the matrix. The 3D sound spectrum contains such information. The spectra responsible for fiber breaking and the occurrence of microcracks are characterized by a course of sharp, high amplitudes in the entire range of the spectrum 1–40 kHz (Figure 8b), whereas the pull-out of fibers and their partial destruction caused an increase in the relative amplitudes, mainly in the area of high frequencies Figure 8c.

The conducted tests indicate that it is possible to accurately identify the destruction processes in cement composites. It should be emphasised, however, that their correct recognition requires an appropriate correlation of the spectra with the individual micro and macro destruction effects.

## 5. Conclusions

It has been indicated that there is a possibility of predicting the occurrence of f_cr_ based on the analysis of sound spectra 3D, the occurring groups of micro-events that precede the end of the load-deflection proportionality area.

The control of micro destruction process before the first crack f_cr_ with the use of acoustic emission (AE) by means of recorded micro-events (increase sumAE) and spectrograms can be used. These analyses should be expanded by adding an interpretation of individual sound spectra (and not the groups of them) and correlated with the load-deflection curve.

The 3D spectra provide a better image of the whole destruction process (particularly with respect to micro-events). The identification of individual events should be correlated with the corresponding individual spectra including the analysis of spectrum in various frequency ranges.

It has been confirmed that the use of spectra with respect to grouped events (destruction processes), in particular in the case of 2D spectra, results in incorrect conclusions if it refers to different events.
